# Slow walking speed and health-related exit from employment among older workers over 5 years of follow-up: evidence from the Health and Employment After Fifty (HEAF) cohort study

**DOI:** 10.1136/bmjopen-2023-081509

**Published:** 2024-07-19

**Authors:** Holly E Syddall, G Ntani, Gregorio Bevilacqua, Elena Zaballa, Stefania D'Angelo, Karen Walker-Bone

**Affiliations:** 1 MRC Versus Arthritis Centre for Musculoskeletal Health and Work, University of Southampton, Southampton, UK; 2 MRC Lifecourse Epidemiology Centre, University of Southampton Faculty of Medicine, Southampton, UK; 3 Monash Centre for Occupational and Environmental Health, Monash University Faculty of Medicine Nursing and Health Sciences, Clayton, Victoria, Australia

**Keywords:** Aging, EPIDEMIOLOGIC STUDIES, PUBLIC HEALTH

## Abstract

**Abstract:**

**Introduction:**

With demographic changes, there is increasing demand for individuals and governments to lengthen working lives. Jobs that are very physically demanding are likely to be more difficult to sustain at older ages. If workers at risk of mismatch of demand and capability could be identified early, there would be opportunities for intervention for health or lifestyle and/or re-training or redeployment.

**Objective:**

To investigate whether self-reported walking speed (a good measure of function in elderly people) predicted health-related job loss (HRJL) longitudinally over 5 years of follow-up among middle-aged workers.

**Design:**

Data came from the Health and Employment After Fifty (HEAF) prospective cohort study of middle-aged people (aged 50–64 years) in UK.

**Setting:**

General population survey (sampling frame was 24 General Practice registers).

**Participants:**

The cohort included 8134 people recruited in 2013–2014. For the current analyses, 5217 people who ever worked and completed at least one follow-up questionnaire were eligible.

**Primary outcome:**

Exit from employment mainly or partly for health reasons (HRJL).

**Results:**

At baseline, very slow walking speed was associated with: obesity, physical inactivity, smoking (men), financial hardship, lower educational attainment and not being in professional occupations. In total, 527 people (10%) reported at least one HRJL during follow-up. After adjustment, the HR for HRJL among men with very slow walking-speed was 4.32, 95% CI 2.72 to 6.87 and among women was 4.47, 95% CI 3.04 to 6.57. After further adjustment for ‘difficulty coping with physical demands at work’, hazards remained doubled in men and women.

**Conclusions:**

Self-reported walking speed could help identify older workers who are at increased risk of HRJL. This could provide opportunities for intervention through optimising health and lifestyle, restricting physical workload, retraining or redeployment. Early appropriate intervention could enable longer working lives and promote healthier, more equal ageing.

**Strengths and limitations of this study**

- Data come from the Health and Employment After Fifty (HEAF) study, a large prospective population cohort study with 5 years of follow-up.
- HEAF has maintained excellent retention rates (71% of those initially recruited) enabling good internal comparisons.
- Our previous analyses on risk factors for health-related job loss in HEAF enabled a considered set of analyses, facilitated by a directed acyclic graph.
- Self-reported walking speed was collected by questionnaires among a range of health and work information so that the risk of responder bias was minimised.

## Introduction

 Life expectancy has increased globally and that, combined with decreasing fertility, has led to rapid demographic change, increasing the proportions of older adults relative to younger people.^1^ In the workplace, relative rates of younger workers have declined, while the number of older people who are no longer economically active is greater after 55 years of age.^2^ Many governments have responded to these challenges by increasing retirement age aiming to address labour shortages, and reduce costs, by prolonging working lives.^3^

Remaining in work to older ages may be difficult for some, particularly, for example, those in physically demanding types of work^4^ like construction.^5^ Peak muscle mass and strength are attained around the fourth decade of life, but thereafter, muscle mass stabilises and then declines, by 1%–2% per year so that more than 50% is lost by age 80 years.^6 7^ Additionally, power and ability to quickly repeat movements attenuate even faster.^8 9^ There is evidence that, although workers age, work demands do not change.^10^ It is therefore a potential concern for individuals, employers and societies if some workers cannot remain in their jobs until older ages. In particular, people who are employed in physically demanding jobs tend to have attained less educational qualifications, so that it can be more difficult for them to retrain or find other sustainable employment.^11^ In turn, this may mean that the most deprived members of the community, who are least well provided for in terms of home ownership^12^ and pensions,^13^ become those most likely to exit employment with a risk of widening health inequalities and increasing the burden on healthcare and welfare support.

It would be advantageous to employers and their workers if people at risk of developing a mismatch between their physical capabilities and the demands of their work could be identified early (when they are still coping at work) so that interventions could be made. For example, among even very elderly people with frailty (a condition of ageing associated with poor physical function which puts individuals at high risk of falls, fractures, hospitalisation and increased mortality), there is evidence that function can be improved with resistance training^14^ and exercise programmes.^15^ In addition to lifestyle interventions, an employer could also provide advice about financial planning and retraining or redeployment opportunities. One recognised predictive marker of physical function among older people is walking speed. It has been shown, for example, to predict dementia,^16^ disability,^17^ mortality^18^ and a range of morbidities including cardiovascular disease.^19^ Although many studies of walking speed have been based on objectively measured walking speed, a systematic review has highlighted the variability of these measures.^20^ Where practical limitations prevent objective measurement, self-reported walking speed has also been shown to reflect functional outcomes,^21 22^ cardiovascular outcomes^23^ and to be strongly associated with measured walking speed.^24^ Given the importance of walking speed as a predictor of mortality and morbidity in older people, it may also be a useful early predictor of declining physical capacity among people in late middle-age. Certainly, we previously found cross-sectionally that individuals reporting markers of frailty (the most important of which was walking-speed) were those most likely to report having stopped working for health reasons.^25^ Therefore, the aim of this study was to investigate the relationship between self-reported walking speed and health-related job loss over 4 years of follow-up among a population sample of older workers aged 50–64 years doing a range of different jobs. Specifically, the research questions were: (a) What are the factors associated with slow walking speed among adults aged 50–64 years? (b) Does self-reported walking speed predict health-related job loss longitudinally? (c) If there is such an association, is there a dose–response relationship?

## Methods

### Population

As described previously, Health and Employment After Fifty (HEAF) is a large population-based cohort of adults in England (aged 50–64 years at baseline).^13^ Briefly, postal questionnaires were mailed to 39 359 adults aged 50–64 years registered with 24 general practices. When they returned their baseline questionnaire, all participants gave written informed consent to participate and to receive annual follow-up questionnaires; this paper utilises data collected from baseline through follow-up 4.

### Questionnaire

The baseline questionnaire enquired about: sociodemographics; lifestyle; employment status and nature and perceptions about working conditions. Questionnaire response categories and groupings for the participant characteristics relevant to this paper are set out in detail in online supplemental appendix 1.

At baseline and at each annual follow-up, participants were also asked ‘Which of the following best describes your walking speed?’ with possible responses on a six-point ordinal scale: unable to walk; very slow; stroll at an easy pace; normal pace; fairly brisk and fast. This self-reported assessment has previously been shown to be a useful marker of timed 3 m walking speed among community-dwelling older people.^23^

At each annual follow-up, participants were asked whether their employment had changed. If relevant, participants reported the dates of leaving and starting a job in the intervening period and stated whether a health problem was mainly or partly the reason for leaving work (referred to here as a ‘health-related job loss’ or HRJL). Participants who changed job were asked the same questions about their current employment as they had been asked about their previous employment. Respondents also provided updated information on walking speed and financial circumstances.

### Patient and public involvement

This cohort study was incepted 2012–2013 and it was not possible to involve patients or the public in the design, or conduct, or reporting, or dissemination plans of our research

### Statistical methods

The principal objective of our analysis was to estimate the effect of slow walking speed (the ‘exposure’) on risk of HRJL (the ‘outcome’) with appropriate adjustment for the role of potential confounders. As previously,^26^ men and women were analysed separately; an analysis strategy decided a priori because work and its social context typically differ between men and women,^27 28^ and previous research has called for investigation of gender differences in risk factors for HRJL.^29^ Analyses were conducted using the Stata statistical software package (release 15). Participant characteristics were summarised using frequency and percentage distributions, means and SD, and medians and IQR ranges.

Walking speed was primarily analysed as a binary variable to minimise sparse data problems: ‘very slow’ (unable to walk or very slow) versus ‘normal’ (stroll at an easy pace, normal pace, fairly brisk or fast). However, the full range of response categories for walking speed was used for supplementary analyses which examined evidence for a ‘dose–response’ relationship between walking speed and HRJL. We used walking speed as first reported (which was baseline for the whole cohort except for 18 participants with missing baseline data and for whom we used their walking speed as reported at 1 year of follow-up) for descriptive purposes. To explore effects of walking speed on HRJL, we used the exposure as a time-varying covariate. Informed by our previous analysis which identified risk factors for HRJL,^26^ we focused on the following characteristics as potential confounders of the association between slow walking speed and risk of HRJL: age; highest educational qualification; self-perceived difficulty managing financially; physical activity; body mass index; smoking status; job satisfaction; coping with the mental demands of the job; coping with the physical demands of the job and self-reported health.

#### Modelling strategy

Confounding variables were selected using a directed acyclic graph (DAG) drawn with the dagitty.net software package (see online supplemental appendix 2Appendix 2); this method enables rigorous identification of confounders by making explicit underlying assumptions about causal associations.^30^ The DAG-implied adjustment set of variables to include in a model to estimate the effect of slow walking speed on HRJL is: age, low education, difficulty managing financially, physical activity, smoking status and obesity. Additionally, identifying the great potential effect of reporting difficulty in copying with physical demands of the job as lying on the causal pathway between walking speed and HRJL, results were also explored after including the relevant variable in the main model. Finally, due to the potential for effects being importantly driven by participants general health, we also adjusted for self-rated health.

#### Survival analysis models

The structure of each follow-up questionnaire enabled respondents to detail the date of leaving a job, and the date of starting a new job (if they started a new job), in the time between subsequent HEAF questionnaires; accordingly, participants could report a maximum of four job exits between HEAF baseline and 4 year follow-up. We configured this information as a multiple-record, multiple-failure survival data set, with time varying covariates for characteristics that changed over time, including walking speed itself.^31^ Each line of this data set represented a period of time during which a respondent was ‘at risk’ of a HRJL (either: the time between two questionnaires during which employment status was unaltered; the time between a questionnaire and a job exit; or the time between the start of a job and the subsequent questionnaire). Each line of the data set recorded the status of the respondent at the end of the time period as: in work; not in work for a health-related reason (a ‘HRJL’); not in work for a reason other than health; not in work for an unspecified reason.

Cox proportional hazards models were used to estimate the effect of slow walking speed as a risk factor for time to first HRJL by fitting the models identified by the DAG and with further adjustment for difficulty in coping with physical demands of the job (as that is believed to lie on the causal pathway). In common with previous studies, we regarded other work outcomes (remaining in employment or job exits for other reasons) as censoring events. HR and 95% CI were estimated using a complete case analysis approach. Tests of the proportional-hazards assumption were based on Schoenfeld residuals and implemented using the *estat phtest* command in Stata. Log–log plots were also used to graphically assess the proportional-hazards assumption.

## Results

### Study sample

8134 participants completed a baseline HEAF questionnaire. 7412 (91%) of these responded to at least one of the four annual follow-ups, among whom 5260 (71%) were in paid employment at some point and of which 5217 (99%, 2515 men and 2702 women) provided sufficient information for inclusion in the survival dataset.

### Walking speed and participant characteristics

Table 1 shows the distribution of first ever self-reported walking speed, which was similar among men and women (p=0.20 for χ^2^ test for independence, 64 (2.5%) men and 82 (3.0%) women reported very slow walking speed). Table 2 shows the distribution of participant characteristics by sex and first reported self-reported walking speed. On average, men and women who reported a very slow walking speed (in comparison with a faster category) were: older; of lower educational attainment; struggling financially; physically inactive; obese; ever smokers (men only); dissatisfied with their job and struggling to cope with the physical and/or the mental demands of work. Taken in combination with our previous work which identified these characteristics as risk factors for HRJL,^26^ these associations confirm the potential role of these characteristics as confounders of the association between slow walking speed and risk of HRJL, and the appropriateness of including them in the DAG (online supplemental appendix 2).

**Table 1:** First reported walking speed by sex among HEAF participants

N (%)	Men (n=2515)	Women (n=2702)
Self-reported walking speed		
Unable to walk	0 (0.0)	0 (0.0)
Very slow	64 (2.5)	82 (3.0)
Stroll at an easy pace	295 (11.7)	320 (11.8)
Normal pace	1127 (44.8)	1144 (42.3)
Fairly brisk	812 (32.3)	940 (34.8)
Fast	217 (8.6)	216 (8.0)

Statistics are frequency and percentage distributions by sex.

For descriptive purposes in this table, walking speed was coded from first available report in the survival analysis file (which was baseline for all but 7 men and 11 women women).

HEAFHealth and Employment After Fifty

**Table 2:** HEAF participant characteristics according to sex and self-reported walking speed

N (%)	Walking speed			
	Men		Women	
	Normal (n=2451)	Very slow (n=64)	Normal (n=2620)	Very slow (n=82)
Sociodemographic				
Age at baseline (years)*	57.8 (42.2)	59.1 (3.9)	57.1 (3.9)	58.1 (4.1)
Highest educational qualification				
No qualifications/school	726 (29.6)	24 (37.5)	933 (35.6)	33 (40.2)
Vocational training certificate	809 (33.0)	26 (40.6)	787 (30.0)	22 (26.8)
University degree/higher	916 (37.4)	14 (21.9)	900 (34.4)	27 (32.9)
How are you managing financially?				
Living comfortably/doing alright/just about getting by	2244 (92.9)	48 (75.0)	2342 (91.3)	56 (70.0)
Finding it difficult/very difficult	172 (7.1)	16 (25.0)	222 (8.7)	24 (30.0)
Lifestyle				
Weekly physical activity				
Some	1818 (84.1)	31 (53.5)	1864 (82.6)	31 (49.2)
None	343 (15.9)	27 (46.6)	393 (17.4)	32 (50.8)
Obesity				
Normal/underweight<25 kg/m^2^	664 (27.8)	10 (15.6)	1143 (44.8)	11 (14.5)
Overweight 25–29.9 kg/m^2^	1178 (49.3)	19 (29.7)	836 (32.8)	16 (21.1)
Obese/severely obese≥30 kg/m^2^	550 (23.0)	35 (54.7)	571 (22.4)	49 (64.5)
Smoking status				
Never	1252 (51.4)	24 (37.5)	1488 (57.4)	43 (53.8)
Ex	909 (37.4)	33 (51.6)	850 (32.8)	25 (31.3)
Current	273 (11.2)	7 (10.9)	256 (9.9)	12 (15.0)
Employment				
Job satisfaction				
Very satisfied/satisfied	2202 (92.6)	54 (85.7)	2422 (94.2)	66 (84.6)
Dissatisfied/very dissatisfied	176 (7.4)	9 (14.3)	148 (5.8)	12 (15.4)
Currently coping with physical demands of the job				
Easily	1745 (73.4)	13 (20.6)	1835 (71.5)	19 (24.1)
Some difficulty or more	633 (26.6)	50 (79.4)	733 (28.5)	60 (76.0)
Currently coping with mental demands of the job				
Easily	1697 (71.5)	36 (57.1)	1726 (67.2)	47 (59.5)
Some difficulty or more	677 (28.5)	27 (42.9)	842 (32.8)	32 (40.5)

Statistics are frequency and percentage distributions within sex and walking speed groups.

For descriptive purposes in this table: walking speed is as coded from first available report in the survival analysis file (which was at baseline for all but 7 men and 11 women women); socio-demographic and lifestyle characteristics are as reported at HEAF baseline. Employment characteristics are coded from the first job reported between HEAF baseline and 4-year year follow-up; this was at baseline for 96% (2425 men and 2592 women women) of the sample, 1-year year follow-up for 2% (50 men and 46 women women), 2-year year follow-up for 1% (26 men, 45 women women), 3-year year follow-up for <1% (8 men, 12 women women) and 4-year year follow-up for <1% (6 men, 7 women women).

* Mean and standard deviationSD.

HEAFHealth and Employment After Fifty

Men with very slow reported walking speed, as compared with those who reported normal walking speed, were more likely to be employed in administrative or secretarial occupations or as process, plant or machine operatives, and were less likely to be employed in professional, associate professional or technical occupations (data not shown). Compared with women reporting normal walking speeds, women with very slow reported walking speed were more likely to be employed in caring, leisure or service occupations or in elementary jobs (data not shown).

### Health-related job loss

Overall, 212 (8.4%) men and 315 (11.7%) women reported leaving a job between baseline and 4 year follow-up because of their health, with only eight of these men and 17 of these women reporting more than one health-related job exit. When asked to attribute their health-related exit, 88 (41.5%) men and 139 (44.1%) women indicated a musculoskeletal cause; 59 (27.8%) men and 120 (38.1%) women indicated a mental health condition; 31 (14.6%) men and 28 (8.9%) women indicated a heart/ lung problem and 65 (30.7%) men and 110 (34.9%) women indicated an ‘other’ health problem (more than one could be nominated). Rates of HRJL per 1000 person-years employed were lower among men (27.6, 95% CI 24.1 to 31.6) than women (38.8, 95% CI 34.7 to 43.3), p<0.001 for sex difference.

### Slow walking speed and risk of health-related job loss

Table 3 shows that crude estimated rates of HRJL by 4 year follow-up were substantially higher among people who reported very slow walking speeds (rates per 1000 person-years employed: men 139.2 (95% CI 92.5 to 209.4); women 196.7 (95% CI 143.1 to 270.3) in comparison with those reporting faster walking speeds (rates per 1000 person-years employed: men 25.0 (95% CI 21.4 to 29.2) and women 33.1 (95% CI 29.0 to 37.8)).

**Table 3:** Associations between self-reported walking speed and risk of 4 year health-related job loss (HRJL), by sex

Self-reported walking speed	Person years employed (1000 s)	HRJLN	HRJL crude rate per1000 person years employed (95% CI)	Model 1HR (95% CI)	Model 2HR (95% CI)	Model 3HR (95% CI)	Model 4HR (95% CI)
Men							
Primary analysis: binary exposure variable							
Very slow	0.165	23	139.2 (92.5 to 209.4)	4.32 (2.72 to 6.87)	2.29 (1.43 to 3.66)	2.09 (1.30 to 3.37)	1.64 (1.02 to 2.65)
Stroll at an easy pace/normal pace/fairly brisk/fast	6.399	160	25.0 (21.4 to 29.2)	Ref	Ref	Ref	Ref
	Supplementary model: all walking speed categories*						
			Very slow	5.40 (3.28 to 8.89)	3.00 (1.80 to 4.98)	2.81 (1.66 to 4.73)	2.14 (1.26 to 3.62)
			Stroll at an easy pace	2.66 (1.80 to 3.92)	1.98 (1.34 to 2.95)	1.95 (1.31 to 2.91)	1.67 (1.11 to 2.50)
			Normal pace	Ref	Ref	Ref	Ref
			Fairly brisk	0.98 (0.67 to 1.42)	1.24 (0.84 to 1.81)	1.14 (0.78 to 1.67)	1.30 (0.88 to 1.90)
			Fast	0.16 (0.04 to 0.64)	0.24 (0.06 to 1.00)	0.20 (0.05 to 0.81)	0.26 (0.06 to 1.09)
Women							
Primary analysis: binary exposure variable							
Very slow	0.193	38	196.7 (143.1 to 270.3)	4.47 (3.04 to 6.57)	2.87 (1.96 to 4.20)	2.32 (1.57 to 3.44)	2.03 (1.38 to 3.00)
Stroll at an easy pace/normal pace/fairly brisk/fast	6.552	217	33.1 (29.0 to 37.8)	Ref	Ref	Ref	Ref
	Supplementary model: all walking speed categories*						
			Very slow	5.08 (3.37 to 7.66)	3.21 (2.12 to 4.85)	2.63 (1.72 to 4.03)	2.19 (1.43 to 3.35)
			Stroll at an easy pace	1.89 (1.35 to 2.65)	1.44 (1.02 to 2.03)	1.39 (0.98 to 2.00)	1.20 (0.85 to 1.70)
			Normal pace	Ref	Ref	Ref	Ref
			Fairly brisk	0.69 (0.49 to 0.96)	0.84 (0.60 to 1.18)	0.79 (0.57 to 1.11)	0.90 (0.64 to 1.27)
			Fast	0.24 (0.10 to 0.59)	0.36 (0.14 to 0.89)	0.30 (0.12 to 0.74)	0.40 (0.16 to 1.00)

HR and 95% CI estimated from Cox proportional hazards models which included walking speed as a time varying covariate and the adjustment factors identified by the directed acyclic graph shown in online supplemental appendix 2.

Model 1: estimates the total effect of self-reported walking speed on risk of HRJL by adjusting for the confounding/biasing effects of age, low education, difficulty managing financially, physical activity, smoking status and obesity.

Model 2: estimates the direct effect of self-reported walking speed on risk of HRJL by adjusting as for model 1 but also for difficulty coping with the physical demands of the job.

Model 3: Ssame as model 1 with extra adjustment for self-rated health.

Model 4: Ssame as model 2 with extra adjustment for self-rated health. Sample sizes [(and number of HRJL events]) analysed were 2,157 [(183]) for HRJL rates and model 1, and 2,133 [(182]) for model 2 for men, and 2,243 [(255]) for HRJL rates and model 1, and 2,221 [(253]) for model 2 for women.

* The results from the supplementary models are shown for illustration of dose-–response effects only. The number of person years employed and the number of HRJL events were low for some of the individual walking speed response categories, which is reflected in comparatively wide confidence intervalCIs for their estimated HRs.

HRJLhealth-related job lossNnumberRefreference category

After adjustment for the potential confounding effects of age, low education, difficulty managing financially, physical activity, smoking status and obesity (model 1), we obtained sizeable estimates for the effect of slow walking speed on increased risk of HRJL among men and women: men (HR 4.32, 95% CI 2.72 to 6.87), women (HR 4.47, 95% CI 3.04 to 6.57). The HRs from model 2, which also adjusted for difficulty coping with the physical demands of the job, were 2.29 (95% CI 1.43 to 3.66) for men and 2.87 (95% CI 1.96 to 4.20) for women; these provide evidence for a robust effect of slow walking speed on risk of HRJL as the effect remained significant beyond adjustment for the effect which operates through difficulty coping with the physical demands of the job. Further adjustment for participants self-rated health (model 3 and model 4) led to a reduction in the effect of walking speed on HRJL, although it still remained statistically significant. Supplementary analyses utilising all the individual response categories for walking speed provided evidence for a dose–response effect of slower walking speed on risk of HRJL among men and women (also presented in table 3).

## Discussion

In this cohort of >5200 working adults aged 50–64 years when recruited, we found that 527 (10%) reported an incident HRJL over 4 years of follow-up and that the risk was predicted by self-reported slow walking speed. Slow walking speed was associated with older age, obesity, ever smoking (men), physical inactivity, lower educational attainment, struggling financially, job dissatisfaction and struggling with physical and mental demands of the job. Men and women with slow walking speed were less likely to be in professional occupations than those reporting normal walking speeds. Women with slow walking speed were more likely to be in caring, leisure or service occupations or in elementary jobs while men were more likely to be in administrative or secretarial occupations or working as process, plant or machine operatives than workers with normal walking speeds. Even after adjustment for confounders, both women and men with self-reported slow walking speed were at fourfold increased risk of a HRJL. Moreover, when additional adjustment was made for difficulty coping with the physical demands of the job (a potential mechanism for these effects), or participants self-reported general health, both men and women remained at a doubled risk of HRJL. The effects of walking speed on the risk of HRJL were dose-related (slower the speed, higher the risk).

The factors we found associated with slow walking speed at baseline were consistent with those reported previously, including smoking, physical inactivity and obesity.^32^ Moreover, more deprived socioeconomic position (SEP), defined here by self-perception of their financial status and educational attainment, has been consistently found associated with slower walking speeds.^33^ Indeed, lower SEP has been found associated with other markers of physical function in older people including grip strength and intrinsic capacity.^1 34 35^ One of the key determinants of adult SEP is occupation and researchers showed that, in the Whitehall study, occupational role carried forward a lasting effect on walking speed after retirement age.^36^ One explanatory hypothesis could be that doing very physically demanding work might increase mechanical loads on joints impairing musculoskeletal health more rapidly and at earlier ages in people doing these types of jobs, with an effect measureable by walking speed. Interestingly, the most common cause attributed for HRJL in the current study was musculoskeletal (in 43% of people). However, when almost 40 000 men and women aged 45–70 years in the Constances cohort study^33^ were investigated, although participants in the lowest/middle SEP had an increased risk of slow walking speed, when they included duration of repetitive work, duration of heavy physical work and lifting heavy weights in their models, they found only a small reduction in the risk estimates obtained (around 10%). They concluded that only a small part of the effects of SEP on walking speed could be explained by lifetime duration of physically demanding work exposures. Exploiting a period of rapid industrialisation in the Irish economy, McCrory *et al* investigated whether social mobility through employment (upwards or downwards) showed a different effect on physical function (walking speed and grip strength) in older age.^37^ Their findings also did not support the hypothesis of greater musculoskeletal deterioration as a result of more deprived SEP accumulating across the life course but rather showed that social mobility in either direction could positively and negatively impact walking speed in later life: walking speed in future more closely resembled that of the SEP in which they ended than in which they started, even though original SEP was strongly associated with final SEP.^37^

It is important to bear in mind that work factors also impact age of retirement and that employers can play an important role in enabling older workers to remain in paid work. We, and others, have found that job dissatisfaction shortens working lives^25 38 39^ and other psychosocial factors including decision authority and appreciation can lengthen them.^40^ There is evidence that, even despite poor self-rated health, workers will remain in work if they feel satisfied with their work.^38^ Thus, there is a role for provision of optimal working conditions in maximising participation of older workers. This may prove more important for retention in some sectors than others, as our results indicated that women with slow walking speed were in caring, leisure and service occupations and elementary jobs, while men with slow walking speed were in administrative/secretarial occupations and process, plant or machine operative roles.

Our findings need to be considered alongside some limitations. First, HEAF is a population cohort assembled all across England which meant that standardised assessment of walking speed was unfeasible. Importantly however self-reported walking speed is also predictive of morbidity and correlates very well with measured walking speed^24^ and our findings show that it predicts the risk of HRJL over 4 years. Moreover, it offers a simple measure that could be used by employers or healthcare providers without additional equipment/space. Our estimates were adjusted for self-rated health, as a measure of general health. However, we cannot exclude the possibility that there would remain some residual confounding attributable to other unidentified long-term conditions, for which we could not control. Nevertheless, considering the strong predictive power of self-rated health for several health outcomes and all-cause mortality, we do not anticipate that additional adjustments for more-specific health measures would eliminate the significant effect of walking speed on HRJL to the extent that it would invalidate our conclusions.

Our choice of grouping ‘stroll at an easy pace’/‘normal pace’/‘fairly brisk’/‘fast’ versus ‘very slow’ was made a priori, aiming to explore a more sensitive measure for screening for individuals at higher risk of HRJL. Sensitivity analysis after grouping ‘very slow’ and ‘stroll’ (the latter also indicating a pace of choice rather than just poor health) together versus ‘normal pace’/‘fairly brisk’/‘fast’ resulted in somewhat attenuated effects. This provides convincing evidence that self-reported slow walking speed is not simply a marker of poor health, and can thus have added value in the measure being used to assess risk of job loss beyond employee’s general health profile.

Second, only around 21% of people invited consented to participate and those who did were older, better educated and wealthier than their equivalents in the general population.^13^ However, our population was representative with regard to employment status, ethnicity and marital status, and included participants throughout England, and from every decile of deprivation. Importantly also, the comparisons presented here are internal, within the cohort and over time, and HEAF has maintained excellent retention rates (71% of those initially recruited) longitudinally. Additionally, everybody in these analyses had ‘survived’ to be in employment at some point during the study period when they were aged at least 50 years, so that they will have some ‘healthy worker’ effect and not be representative of a general population sample. However, to address the specific questions posed here, they were the correct selected sample. The study also benefits from the use of longitudinal data from a prospective cohort with a large sample size.

There are complex interrelationships between work and health and the effects are bidirectional as poor health causes job loss, while poor working conditions cause poor health. Physically demanding work contributes to health inequalities^41^ throughout the life course but it appears that these effects may be brought into even sharper relief among older workers. Although extending working lives is a policy priority in many countries, the effect of these policies may be felt unevenly and widen health inequalities such that those most vulnerable as a result of poorer health, more physically demanding work and lowest educational attainment are unable to remain working and struggle most to find alternative employment.^11^ Our findings suggest that monitoring the health of older workers, using simple measures like self-reported walking speed could identify those at highest risk. It would also be appropriate to consider reducing the physical workload for all older workers in order to maximise the possibility of retaining them in work. Alongside policy initiatives to extend working lives, governments may need to offer retraining and redeployment opportunities or alternatively, take a more nuanced approach to age of eligibility for pensions, considering the nature of work.

In conclusion, self-reported slow walking speed was associated with at least a doubling in the risk of incident HRJL over 4 years of follow-up among older workers. This simple measure could be part of a strategy used to identify workers at high risk and intervene with improved working conditions, optimising health and/or considering opportunities for retraining or redeployment.

## supplementary material

10.1136/bmjopen-2023-081509 online supplemental file 1

10.1136/bmjopen-2023-081509 online supplemental file 2

## Data Availability

Data are available upon reasonable request.
