# Supplementary material for: Slow walking speed and health-related exit from employment among older workers over 5 years of follow-up: evidence from the Health and Employment After Fifty (HEAF) cohort study
Source: BMJ Open. 2024 Jul 19;14(7):e081509. doi: 10.1136/bmjopen-2023-081509 (PMC11288146; doi:10.1136/bmjopen-2023-081509)
Supplement: online supplemental file 1 [file bmjopen-14-7-s001.pdf]

Appendix 1. HEAF questions, response categories, coded analysis variables and reference categories (in italics).

**Question**	**Response categories**	**Further coding for analysis**
Do you have any of the following qualifications?	o/gcse levels; a levels; vocational cert; university degree; higher professional qualification	no qualifications/school level vs *vocational training certificate* vs university degree/higher
How well do you feel you are managing financially these days?	*living comfortably/doing alright/just about getting by*; finding it difficult/very difficult to make ends meet	
In an average week, outside work, roughly how many hours would you spend doing the following activities?	hours per week physical activities sufficient to make you hot or sweaty	*any hours* vs no hours
Please give your height and your weight	height reported in feet and inches, or centimetres<br>weight reported in stones and pounds, or kilograms	Converted to height in metres and weight in kilograms. BMI in kg/m^2 calculated as height divided by the square of weight, and categorised as: normal/underweight $<25kg/m^2$; *overweight 25-29.9kg/m²*; $\geq 30kg/m^2$ (obese/severely obese).
Have you ever smoked regularly?<br>If yes, do you still smoke regularly?	no; yes response categories for both questions	Smoker status coded as: *never*; ex/current
In general would you say your health is?	excellent; very good; good; fair; poor	‘at least good’ vs ‘fair/poor’
Which of the following best describes your walking speed?	unable to walk; very slow; stroll at an easy pace; normal pace; fairly brisk; fast	‘unable to walk/very slow’ vs *‘stroll at an easy pace/normal pace/fairly brisk/fast’*

The following items were completed by employed or self-employed people only

**Question**	**Response categories**	**Further coding for analysis**
How satisfied are you with your job as a whole?	very satisfied; satisfied; dissatisfied; very dissatisfied	*very satisfied/satisfied vs* dissatisfied/very dissatisfied
Currently, how well do you cope with the physical demand of the job?	easily; with some difficulty; with great difficulty; not coping	*easily* vs at least some difficulty
Currently, how well do you cope with the mental demand of the job?	easily; with some difficulty; with great difficulty; not coping	*easily* vs at least some difficulty
