# Supplementary material for: Slow walking speed and health-related exit from employment among older workers over 5 years of follow-up: evidence from the Health and Employment After Fifty (HEAF) cohort study
Source: BMJ Open. 2024 Jul 19;14(7):e081509. doi: 10.1136/bmjopen-2023-081509 (PMC11288146; doi:10.1136/bmjopen-2023-081509)

Appendix 2. Directed acyclic graph for the effect of slow walking speed on health-related job loss (HRJL).

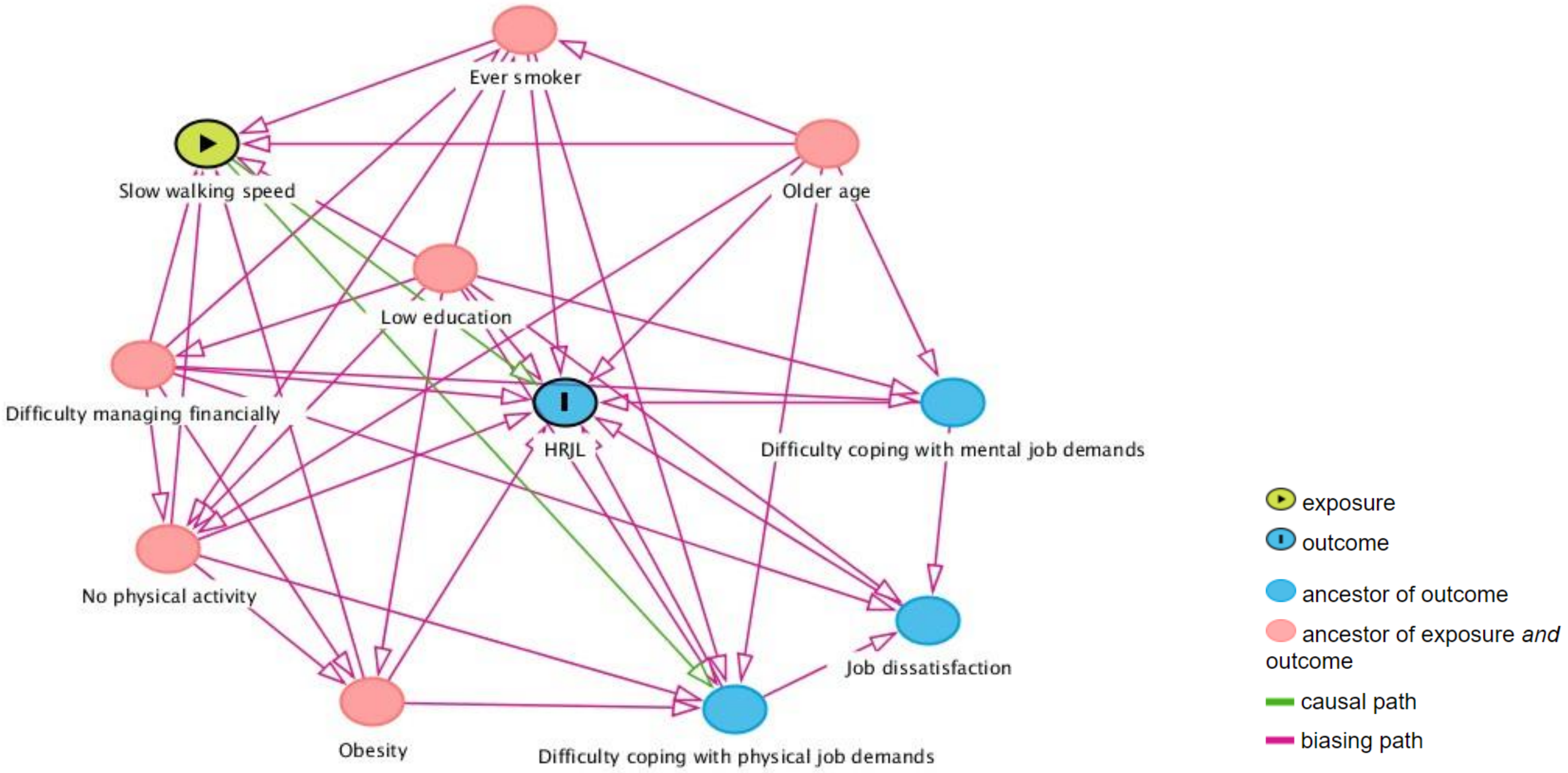

Supplement: online supplemental file 2 [file bmjopen-14-7-s002.pdf]
