# Supplementary material for: Slow walking speed and health-related exit from employment among older workers over 5 years of follow-up: evidence from the Health and Employment After Fifty (HEAF) cohort study
Source: BMJ Open. 2024 Jul 19;14(7):e081509. doi: 10.1136/bmjopen-2023-081509 (PMC11288146; doi:10.1136/bmjopen-2023-081509)
Supplement: Supplementary file 3 [file bmjopen-2023-081509.reviewer_comments.pdf]

PEER REVIEW HISTORY

BMJ Open publishes all reviews undertaken for accepted manuscripts. Reviewers are asked to complete a checklist review form (**http://bmjopen.bmj.com/site/about/resources/checklist.pdf**) and are provided with free text boxes to elaborate on their assessment. These free text comments are reproduced below.

ARTICLE DETAILS

**TITLE (PROVISIONAL)**	SLOW WALKING SPEED AND HEALTH-RELATED EXIT FROM EMPLOYMENT AMONG OLDER WORKERS OVER 5 YEARS OF FOLLOW-UP: EVIDENCE FROM THE HEALTH AND EMPLOYMENT AFTER FIFTY (HEAF) COHORT STUDY
**AUTHORS**	Syddall, Holly E; Ntani, G; Bevilacqua, Gregorio; Zaballa, Elena; D'Angelo, Stefania; Walker-Bone, Karen

VERSION 1 – REVIEW

**REVIEWER**	Zhang, James<br>University of Chicago, Medicine
**REVIEW RETURNED**	16-Dec-2023

**GENERAL COMMENTS**	This study aimed to investigate whether self-reported walking speed predicted health-related job loss (HRJL) longitudinally over 5 years of follow-up among middle-aged workers using a dataset from the Health and Employment After Fifty (HEAF) prospective cohort study of middle-aged people (aged 50-64 years) in UK. This is topically important for public health and maintaining productivity among the middle-aged population. In the meantime, clarifications are needed for conceptualization, competing risk, methods, and discussion/limitation,<br><br>Conceptualization: the authors stated that "Participants were also asked 'Which of the following best describes your walking speed?' with possible responses on a six-point ordinal scale: unable to walk; very slow; stroll at an easy pace; normal pace; fairly brisk; fast" as part of baseline questionnaires (Line 148-149), and "At each annual follow-up, participants were asked whether their employment had changed. If relevant, participants reported the dates of leaving and starting a job in the intervening period and stated whether a health problem was mainly or partly the reason for leaving work (referred to herein as a 'health-related job loss' or HRJL)." (154-157). The baseline response was then used to predict the outcome of HRJL controlling for other risk factors including the time-varying covariate of walking speed itself. The authors aimed to derive the effect of baseline walking speed on the HRJL during the follow-up period. Since this is a longitudinal study with 5-year follow-up, and the baseline questionnaire was on an undefined recall timeframe ("In an average week, outside work, roughly how many hours would you spend doing the following activities? "Appendix I), it's unclear if the HRJL should be attributed to the walking at the baseline or during the follow-up

	period. A well-thought-out discussion of the baseline questionnaire, the potential impact of the exercise routine during the follow-up period, and their relative effect size of these two will help readers to better understand the significance, limitation, and validity of the research findings.<br>Competing risk: death/dropouts. The authors stated that “8,134 participants completed a baseline HEAF questionnaire. 7,412 (91%) of these responded to at least one of the four annual follow-ups.” (Line 219-220). However, Table 1 showed the total N as 5217 (2515+2702). Please clarify the discrepancy. In addition, were there any persons deceased during the study period? For middle-aged people with serious underlying conditions, mortality is a competing risk for staying in the jobs (and answering any follow-up questionnaires). A careful examination of death and dropouts will provide insight into the potential bias of the estimation.<br>Method. The authors stated that “We configured this information as a multiple-record, multiple-failure survival dataset, with time varying covariates for characteristics that changed over time, including walking speed itself (30). Each line of this dataset represented a period of time during which a respondent was ‘at risk’ of a health-related job loss (either: the time between two questionnaires during which employment status was unaltered; the time between a questionnaire and a job exit; or the time between the start of a job and the subsequent questionnaire). Each line of the dataset recorded the status of the respondent at the end of the time period as: in work; not in work for a health-related reason (a ‘HRJL’); not in work for a reason other than health; not in work for an unspecified reason.” (Line 195-202). It’s unclear what “a multiple-record, multiple-failure survival dataset” means. Is it a repeated measurement that each respondent had multiple entries hence multiple endpoints? Please clarify. If so, how was the correlation matrix specified in the survival analysis? Please also cite relevant references to support such an approach.

**REVIEWER**	Merom, Dafna<br>Western Sydney University, School of Health Science
**REVIEW RETURNED**	17-Dec-2023

**GENERAL COMMENTS**	In this manuscript, the investigators wished to assess whether a simple question reflecting an individual’s perception of walking speed can predict health-related job loss (HRJL). The rationale for examining this question is that, if this relationship holds, then using such a simple measure can identify older employees at risk of losing their jobs prematurely and intervening. The authors found that those who reported walking “very slow” had 4 times the risk of HRJL, and this was true for men and women.<br>The methods are well described, and the supplementary table 3 contributes further to the clarity of the findings, showing that the risk of HRJL was significant, albeit attenuated, also for those reported strolling compared to normal pace.<br><br>My main concern is the lack of adjustment to health status. The slow walk may be a marker for existing serious health conditions such (eg. heart disease, all type of arthritis, chronic pain, poor mental health etc). Even a simple question “How would you rate

	your health?" excellent, good, fair, poor, very poor" could have eliminated the association between walking pace and HRJL. I would be much more convinced in the findings had the authors included in their model adjustment to existing health conditions, or to a simple self-rated health question and then demonstrate that walking speed remained as a predictor.<br>Can the author refer to why health status (objective or subjective) was not included in the model? If they did not have such information, they must include it as a discussion point – it is a major confounder that was ignored. The adjustment to "difficulty coping with physical demands of the job" IS NOT THE SAME as adjusting to health conditions or self-reporting health status.<br>Also the manipulation of the physical activity measure as "none versus some" is also inviting residual confounding this categorisation is not sensitive enough – why not to model hours per week if this is how the question was asked?<br><br>Minor comments:<br>Introduction: in the first paragraph the authors wrote: "whilst the number of older people who are no longer economically active but need to be supported increases, effectively growing the financial burden on societies.<br>Can the author provide a reference for this assertion, is it worldwide or in the UK only?<br><br>Methods- I think it is important to clarify why 'very slow' and 'stroll 'were not grouped under one category against all others in the main analysis, it will certainly attenuate the association but will be more convincing that this category is not a marker of poor health considering strolling can be a pace of choice. The validation study (Ref 23) shows these two categories are below 0.91m/s, within the speed zone that predicted many health hazards, such as falls and mortality, for older adults.<br>Also, it will be important to acknowledge that the cohort in the validation study (Ref 23) was much older than the sample of the HEAF; can the authors comment on how this age discrepancy can affect the results? Is this could heighten the finding given that for 50-61 years old person, walking very slowly may indicate a real health problem. It will be good to include the corresponding values from the validation study in the discussion as it is of interest to clinicians and other health professional.

VERSION 1 – AUTHOR RESPONSE

Reviewer: 1
Dr. James Zhang, University of Chicago
Comments to the Author:
This study aimed to investigate whether self-reported walking speed predicted health-related job loss (HRJL) longitudinally over 5 years of follow-up among middle-aged workers using a dataset from the Health and Employment After Fifty (HEAF) prospective cohort study of middle-aged people (aged 50-64 years) in UK. This is topically important for public health and maintaining productivity among the

middle-aged population. In the meantime, clarifications are needed for conceptualization, competing risk, methods, and discussion/limitation,

Thank you for acknowledging the importance of the study and research questions.

Conceptualization: the authors stated that "Participants were also asked 'Which of the following best describes your walking speed?' with possible responses on a six-point ordinal scale: unable to walk; very slow; stroll at an easy pace; normal pace; fairly brisk; fast" as part of baseline questionnaires (Line 148-149), and "At each annual follow-up, participants were asked whether their employment had changed. If relevant, participants reported the dates of leaving and starting a job in the intervening period and stated whether a health problem was mainly or partly the reason for leaving work (referred to herein as a 'health-related job loss' or HRJL)." (154-157). The baseline response was then used to predict the outcome of HRJL controlling for other risk factors including the time-varying covariate of walking speed itself. The authors aimed to derive the effect of baseline walking speed on the HRJL during the follow-up period. Since this is a longitudinal study with 5-year follow-up, and the baseline questionnaire was on an undefined recall timeframe ("In an average week, outside work, roughly how many hours would you spend doing the following activities? "Appendix I), it's unclear if the HRJL should be attributed to the walking at the baseline or during the follow-up period.

Thank you very much for this comment. We apologise for any lack of clarity on our methods and how data were collected and used in this analysis. To clarify, walking speed was measured at baseline and at each of the 4 years of follow-up. For descriptive purposes only, we used walking speed as firstly reported (which was baseline for the whole cohort except 18 participants with missing baseline data and for whom we used their walking speed as reported at 1 year of follow-up) to describe this exposure measure among men and women in our study (Table 1). Similarly, we used walking speed as coded from first available report in the survival analysis file (which was at baseline for all but 18 participants) to describe participants' characteristics according to their sex and whether they self-reported 'normal' or 'very slow' walking speed (Table 2). So, the fixed exposure measure was used for descriptive purposes only. For the main survival analysis exploring the effect of slow walking speed on health-related job loss, we used the time-varying exposure measure, instead of the baseline fixed one (see Table 3 footnote: "Hazard ratios and 95%CI estimated from Cox proportional hazards models which included walking speed as a time varying covariate"). Also, the outcome event occurred at different time points during follow-up for different participants, and not specifically at 5 years, as the reviewer suggests. Data were collected prospectively, and we estimated the effect of yearly reported walking speed (time-varying exposure) on risk of job loss on health grounds. As such, we believe that our methodological approach is unlikely to give rise to recall bias, and it is likely that any associations explored through our analysis are likely to be causally linked. However, to avoid future readers' confusion, we have added text to clarify our methods.

Line 150 in the revised manuscript: "*At baseline and at each annual follow-up, participants were also asked…*".

Lines 179-183 in the revised manuscript: "*We used walking speed as firstly reported (which was baseline for the whole cohort except for 18 participants with missing baseline data and for whom we used their walking speed as reported at 1 year of follow-up) for descriptive purposes. To explore effects of walking speed on HRJL, we used the exposure as a time-varying covariate*."

A well-thought-out discussion of the baseline questionnaire, the potential impact of the exercise routine during the follow-up period, and their relative effect size of these two will help readers to better understand the significance, limitation, and validity of the research findings.

The reviewer makes a very interesting point regarding the interplay between walking speed and exercise routines and their relative effects on health-related job loss. Our primary aim in this paper was to explore the effect of walking speed, as the most important marker of frailty, on health-related job loss. Fitness is likely to be associated with walking speed, yet, its effect/association with HRJL is not established, and as such the potential of confounding effect cannot be clearly justified. We did however repeat the analysis after adjusting for self-reported engagement with physical activity, and the association between walking speed and health-related job loss remained unchanged (data not shown).

Competing risk: death/dropouts. The authors stated that "8,134 participants completed a baseline HEAF questionnaire. 7,412 (91%) of these responded to at least one of the four annual follow-ups." (Line 219-220). However, Table 1 showed the total N as 5217 (2515+2702). Please clarify the discrepancy.

As reported in the first paragraph of the results "8,134 participants completed a baseline HEAF questionnaire. 7,412 (91%) of these responded to at least one of the four annual follow-ups, amongst whom 5,260 (71%) were in paid employment at some point and of which 5,217 (99%, 2,515 men and 2,702 women) provided sufficient information for inclusion in the survival dataset." (lines 228-231 in the revised manuscript).

In addition, were there any persons deceased during the study period? For middle-aged people with serious underlying conditions, mortality is a competing risk for staying in the jobs (and answering any follow-up questionnaires). A careful examination of death and dropouts will provide insight into the potential bias of the estimation.

The point that the reviewer makes is valid. Yet, our HEAF cohort is not currently linked with mortality data. We however can make the point that HEAF participants have been very engaged with this survey and are in regular contact via telephone, email and letters informing us of changes in personal circumstances. [In many cases both eligible members of a household are participating]. Only very few participants have notified us of the death of other participants in the HEAF study, making it difficult to produce any insightful analyses on such small numbers.

Method. The authors stated that “We configured this information as a multiple-record, multiple-failure survival dataset, with time varying covariates for characteristics that changed over time, including walking speed itself (30). Each line of this dataset represented a period of time during which a respondent was ‘at risk’ of a health-related job loss (either: the time between two questionnaires during which employment status was unaltered; the time between a questionnaire and a job exit; or the time between the start of a job and the subsequent questionnaire). Each line of the dataset recorded the status of the respondent at the end of the time period as: in work; not in work for a health-related reason (a ‘HRJL’); not in work for a reason other than health; not in work for an unspecified reason.” (Line 195-202). It’s unclear what “a multiple-record, multiple-failure survival dataset” means. Is it a repeated measurement that each respondent had multiple entries hence multiple endpoints? Please clarify. If so, how was the correlation matrix specified in the survival analysis? Please also cite relevant references to support such an approach.

The observation time for each participant in the study was broken down into multiple records as follows: Each participant had at least as many records (lines in the survival analysis file) as questionnaires completed (i.e. follow-ups and one baseline). If a participant did not have any job changes, then their records were not further split into sub-records. If a participant had a job change (i.e. changing from one job reported at a time point T1 to another job reported at the next time point T2), their corresponding observation time ‘T1-T2’ (i.e. record corresponding to ‘T1-T2’ period) was split into 2 records, the first being ‘T1-job exit’ and the second being ‘new job start – T2’. The term “multiple-record” refers to the repeated measures due to the follow-ups and further to the job changes.

As described in the methods, each record corresponded to a time period and HRJL represented participants job status at the end of that period. If the end of any given period/record was the time of the questionnaire, then HRJL was marked as “in work”, while if the end of any given period was the time of a job exit, HRJL was marked either as “not in work for a health-related reason” or “not in work for a reason other than health” or “not in work for an unspecified reason”. As several participants changed more than one jobs over follow-up in the study, some experienced the outcome of interest (HRJL) more than once. As such, our survival file is further characterised as “multiple-failure” survival data file.

This analytical approach has been used in our HEAF published research outputs listed below:

Syddall HE, D'Angelo S, Ntani G, Stevens M, Harris EC, Linaker CH, Walker-Bone K. Work participation and risk factors for health-related job loss among older workers in the Health and Employment after Fifty (HEAF) study: Evidence from a 2-year follow-up period. PLoS One. 2020 Sep 17;15(9):e0239383. doi: 10.1371/journal.pone.0239383. PMID: 32941536; PMCID: PMC7498069. *

Bevilacqua G, D'Angelo S, Ntani G, Stevens M, Linaker CH, Walker-Bone K. Shift work amongst older UK workers and job exit. Occup Med (Lond). 2021 Dec 24;71(9):429-438. doi: 10.1093/occmed/kqab131. PMID: 34693446.

D'Angelo S, Syddall H, Ntani G, Harris EC, Linaker C, Cooper C, Stevens M, Walker-Bone K. How does job dissatisfaction interact with self-rated health in determining the risk of health-related job loss? Prospective findings from the Health and Employment After Fifty (HEAF) study. Occup Environ Med. 2021 Jan;78(1):36-42. doi: 10.1136/oemed-2020-106600. Epub 2020 Sep 11. PMID: 32917741; PMCID: PMC7116481.

Walker-Bone K, D'Angelo S, Linaker CH, Stevens MJ, Ntani G, Cooper C, Syddall HE. Morbidities among older workers and work exit: the HEAF cohort. Occup Med (Lond). 2022 Oct 18;72(7):470-477. doi: 10.1093/occmed/kqac068. PMID: 35904117; PMCID: PMC9578672.

Reviewer: 2
Dr. Dafna Merom, Western Sydney University
Comments to the Author:
In this manuscript, the investigators wished to assess whether a simple question reflecting an individual's perception of walking speed can predict health-related job loss (HRJL). The rationale for examining this question is that, if this relationship holds, then using such a simple measure can identify older employees at risk of losing their jobs prematurely and intervening. The authors found that those who reported walking "very slow" had 4 times the risk of HRJL, and this was true for men and women.
The methods are well described, and the supplementary table 3 contributes further to the clarity of the findings, showing that the risk of HRJL was significant, albeit attenuated, also for those reported strolling compared to normal pace.

Thank you for this coment.

My main concern is the lack of adjustment to health status. The slow walk may be a marker for existing serious health conditions such (eg. heart disease, all type of arthritis, chronic pain, poor mental health etc). Even a simple question "How would you rate your health?" excellent, good, fair, poor, very poor" could have eliminated the association between walking pace and HRJL. I would be much more convinced in the findings had the authors included in their model adjustment to existing health conditions, or to a simple self-rated health question and then demonstrate that walking speed remained as a predictor.

Can the author refer to why health status (objective or subjective) was not included in the model? If they did not have such information, they must include it as a discussion point – it is a major confounder that was ignored. The adjustment to "difficulty coping with physical demands of the job" IS NOT THE SAME as adjusting to health conditions or self-reporting health status.

Also the manipulation of the physical activity measure as "none versus some" is also inviting residual confounding this categorisation is not sensitive enough – why not to model hours per week if this is how the question was asked?

Thank you for this insightful comment. The main purpose of this study was to assess whether the measure of walking speed can be used for predicting HRJL. We agree with the reviewer that the effect of general health is one that could potentially alter the associations reported and indeed the conclusions. As such, the main effects should be "corrected" for general health. After adjusting for general health (assessed through the question "In general would you say your health is: excellent – very good – good – fair – poor"), the main effects of walking speed on HRJL were attenuated, yet they remained significant, confirming validity of our findings. We believe that these adjusted effects add interpretation value to our findings. Thus, we have added another 2 columns in Table 3 to display the results adjusted for general health. We have also added description of the self-rated health measure in Appendix 1, and description in the statistical analysis and results of the manuscript.

Line 187: Added "*and self-reported health*"

Line 197-198: Added "*Lastly, due to the potential for effects being importantly driven by participants general health, we also adjusted for self-rated health.*"

Line 274-275: Added "*Further adjustment for participants self-rated health (model 3 and model 4) led to a reduction in the effect of walking speed on HRJL, although it still remained statistically significant.*"

Table 3 footnotes: Added "*Model 3: Same as model 1 with extra adjustment for self-rated health*

Model 4: Same as model 2 with extra adjustment for self-rated health"

Regarding "difficulty in coping with physical demands of the job", our intention was not to use this as a measure of health, as the reviewer hypothesised. In our thinking through the potential mechanisms connecting slow walking speed and health-related job exit, we identified the possibility of "difficulty in coping with physical demands of the job" lying on the causal pathway, i.e. slow walking speed indicating frailty could lead to people's incapacity to cope with physical demands of their job and thus leaving their job on health grounds. As such, "difficulty in coping with physical demands of the job" could have an important mediating effect, the exploration of which could shed light on the reported associations.

Minor comments:
Introduction: in the first paragraph the authors wrote: "whilst the number of older people who are no longer economically active but need to be supported increases, effectively growing the financial

burden on societies.
Can the author provide a reference for this assertion, is it worldwide or in the UK only?

Thank you for your comment. The problem of economic inactivity among older workers, with potential implications on societies expands beyond the UK but is currently most marked in high-income countries (Japan has the highest rate). We agree that this statement should be referenced. We have now added the following reference:

OECD 2024. Labour force participation rate (indicator). Accessed from: doi:10.1787/8a801325-en (accessed 19th February 2024)

Methods- I think it is important to clarify why 'very slow' and 'stroll 'were not grouped under one category against all others in the main analysis, it will certainly attenuate the association but will be more convincing that this category is not a marker of poor health considering strolling can be a pace of choice.

Our decision of keeping the 'very slow' in a separate category was made on the basis of separating the most 'severe' category from the rest, potentially resulting in a more sensitive measure for screening individuals at higher risk of HRJL. Following the reviewer's suggestion, we have repeated the analysis after grouping 'very slow' and 'stroll' together against 'normal pace'/ 'Fairly brisk' / 'Fast'. As the Reviewer anticipated, the effects were somewhat attenuated, yet they remained significant, providing convincing evidence that slow walking speed is not simply a marker of poor health, and can thus have added value in the measure being used to assess risk of job loss beyond employee's general health profile. We have added the following text:

Line 331-337: "*Our choice of grouping 'stroll at an easy pace'/'normal pace'/'fairly brisk'/'fast' versus 'very slow' was made a priori, aiming to explore a more sensitive measure for screening individuals at higher risk of HRJL. Sensitivity analysis after grouping 'very slow' and 'stroll' (the latter also indicating a pace of choice rather than just poor health) together versus 'normal pace'/'fairly brisk'/'fast' resulted in the effects of self-rated walking speed being somewhat attenuated. This provides convincing evidence that self-reported slow walking speed is uncommon in this age group and when reported adds value in gauging the risk of health-related job loss, extending beyond employee's general health status.* "

The validation study (Ref 23) shows these two categories are below 0.91m/s, within the speed zone that predicted many health hazards, such as falls and mortality, for older adults.
Also, it will be important to acknowledge that the cohort in the validation study (Ref 23) was much older than the sample of the HEAF; can the authors comment on how this age discrepancy can affect the results? Is this could heighten the finding given that for 50-61 years old person, walking very slowly may indicate a real health problem. It will be good to include the corresponding values from the

validation study in the discussion as it is of interest to clinicians and other health professional.

Indeed, slower walking speed in a younger population compared to an older one is likely to be more strongly associated with poor health. Yet, our results showed that the association between walking speed and HRJL was robust to adjustment for general health. Additionally, as our HEAF participants were aged 50-64 at baseline, their mean age at follow-up (in the survival file defined as either HRJL event or end of observation time) was 59.8 (range: 50.4-69.7), while mean age in the validation cohort was 67 (range: 61-73). We consider this difference in the age distribution rather small to be importantly biasing our reported associations.

Reviewer: 1
Competing interests of Reviewer: None.

Reviewer: 2
Competing interests of Reviewer: Non declared

VERSION 2 – REVIEW

**REVIEWER**	Zhang, James<br>University of Chicago, Medicine
**REVIEW RETURNED**	09-Mar-2024

**GENERAL COMMENTS**	Thanks for the opportunity to review the revised manuscript. While this is an improved manuscript with better clarity, the concern remains if the health-related employment exit was attributable to walking exercise, of both speed of walking and health-related employment exit were due to other underlining structure unobserved in the study. For example, the onset and/or progression of chronic conditions might have affected both. The authors added general health as a covariate in the revised manuscript, but many other factors particularly the chronic conditions that could have affected both walking speed and health related employment exit were not controlled for. The causal path is a complex one beyond the discussion the authors have offered, and a well-thought-out of discussion of such complexity and implications of the omission of other important factors will help reader to better understand the strength and limitations of the study. A more cautious tone in the Conclusion in the Abstract is needed.

VERSION 2 – AUTHOR RESPONSE

Reviewer: 1

Dr. James Zhang, University of Chicago

Comments to the Author:

Thanks for the opportunity to review the revised manuscript. While this is an improved manuscript with better clarity, the concern remains if the health-related employment exit was attributable to walking exercise, of both speed of walking and health-related employment exit were due to other underlining structure unobserved in the study. For example, the onset and/or progression of chronic conditions might have affected both. The authors added general health as a covariate in the revised manuscript, but many other factors particularly the chronic conditions that could have affected both walking speed and health related employment exit were not controlled for. The causal path is a complex one beyond the discussion the authors have offered, and a well-thought-out of discussion of such complexity and implications of the omission of other important factors will help reader to better understand the strength and limitations of the study. A more cautious tone in the Conclusion in the Abstract is needed.

Thank you for this comment. We appreciate the concern about residual confounding in the associations presented in this study, even after adjusting for poor self-rated health (SRH). However, SRH has been repeatedly shown to be highly predictive of a full range of health outcomes and has been even shown to be a stronger predictor of all-cause mortality than other instruments designed for this purpose (Ganna Andrea, Ingelsson Erik. 5 year mortality predictors in 498 103 UK Biobank participants: a prospective population-based study. The Lancet. 2015;386(9993):533–540. doi: 10.1016/S0140-6736(15)60175-1). As such, we believe it is unlikely that any more specific measure of health would better explain the association between walking speed and HRJL reported in our study over and above what has been explained by SRH, or not sufficiently to invalidate our conclusions. Despite this, recognising the small possibility of residual confounding that might further explain the reported effects, we have added the following statement to the limitations of the study in the Discussion:

“Our estimates were adjusted for self-rated health, as a measure of general health. However, we cannot exclude the possibility that there would remain some residual confounding attributable to other unidentified long-term conditions, for which we could not control. Nevertheless, considering the strong predictive power of self-rated health for several health outcomes and all-cause mortality, we do not anticipate that additional adjustments for more-specific health measures would eliminate the significant effect of walking speed on HRJL to the extent that it would invalidate our conclusions”.

We have also re-phrased the conclusive statement in the Abstract, as below:

Self-reported walking speed could help identify older workers who are at increased risk of HRJL. This could provide opportunities for intervention through optimising health and lifestyle, restricting physical workload, re-training or re-deployment. Early appropriate intervention could enable longer working lives and promote healthier, more equal ageing.

Reviewer: 1

Competing interests of Reviewer: None

VERSION 3 – REVIEW

**REVIEWER**	Zhang, James<br>University of Chicago, Medicine
**REVIEW RETURNED**	17-Jun-2024

**GENERAL COMMENTS**	The authors were responsive to the reviewer's comments. This is a manuscript with improved clarity and tighter conclusion.

VERSION 3 – AUTHOR RESPONSE
